# Do artisanal fishers perceive declining migratory shorebird populations?

**DOI:** 10.1186/s13002-016-0087-x

**Published:** 2016-03-03

**Authors:** Luciano Pires Andrade, Horasa Maria Lima Silva-Andrade, Rachel Maria Lyra-Neves, Ulysses Paulino Albuquerque, Wallace Rodrigues Telino-Júnior

**Affiliations:** Graduate Program in Ethnobiology and Nature Conservation-PPGEtno, Federal Rural University of Pernambuco, Rua Dom Manoel de Medeiros, s/n, Dois Irmãos, 52171-900 Recife, Pernambuco Brazil; Federal Rural University of Pernambuco, Garanhuns Campus, Av. Bom Pastor, s/n, Boa Vista, 55292-270 Garanhuns, Pernambuco Brazil; Department of Biology, Laboratory of Ecology and Evolutivon of Social-Ecological Systems (LEA), Federal Rural University of Pernambuco, Rua Dom Manoel de Medeiros, s/n, Dois Irmãos, 52171-900 Recife, Pernambuco Brazil

**Keywords:** Local ecological knowledge, Ethnobiology, Human ecology, Ethno-ornithology

## Abstract

**Background:**

This paper discusses the results of ethno-ornithological research conducted on the local ecological knowledge (LEK) of artisanal fishers in northeast Brazil between August 2013 and October 2014.

**Methods:**

The present study analyzed the LEK of 240 artisanal fishermen in relation to Nearctic shorebirds and the factors that may be affecting their populations. We examined whether differences occurred according to the gender and age of the local population. The research instruments included semi-structured and check-list interviews.

**Results:**

We found that greater knowledge of migratory birds and the areas where they occur was retained by the local men compared with the local women. Half of the male respondents stated that the birds are always in the same locations, and most of the respondents believed that changes in certain populations were caused by factors related to habitat disturbance, particularly to increases in housing construction and visitors to the island. The main practices affecting the presence of migratory birds mentioned by the locals were boat traffic and noise from bars and vessels. According to the artisanal fishermen, the population of migratory birds that use the area for foraging and resting has been reduced over time.

**Conclusions:**

Changes in the local landscape related to urbanization and tourism are most likely the primary causes underlying the reduced migratory shorebird populations as reported by local inhabitants. Thus, managing and monitoring urbanization and tourism are fundamental to increasing the success of the migration process and improving the conservation of migratory shorebird species.

## Background

The continued growth of human populations along coastal zones has intensified the extraction of natural resources, increased environmental pressures related to recreational activities, and acts as a primary driver of natural habitat degradation, loss, and fragmentation [1–4]. Approximately half of the world’s major urban centers and two-thirds of the global population are found within 60 km of a coastline [5, 6], which has fundamental implications for the long-term environmental conditions of coastal areas [4, 7]. The global settlement pattern of human societies reinforces the degradation of coastal environments and the negative impacts on their natural resources; thus, coastal ecosystems are among the planet’s most threatened regions [6, 8], particularly for species that depend on these habitats, such as migratory shorebirds.

Aquatic birds, particularly migratory shorebirds, are highly dependent on coastal areas during their migration [9–11]. Because these birds return to the same overwintering sites after breeding in the northern hemisphere [12], the quality of these habitats is fundamental to their annual life cycle and the long-term survival of their populations [13–16]; thus, these birds must be able to return to their breeding grounds year after year [12].

South America is visited by approximately 2.9 million migratory shorebirds each year [14, 15, 17], and the northeastern coast of the continent includes a number of areas that are considered key sites for migratory shorebirds that follow the Atlantic migratory route [16, 18–21]. These overwintering sites are used by the birds to recover from their long migratory flights and to forage for food to accumulate body fat for the return journey to the Northern Hemisphere [22]. Therefore, the quality of these sites is fundamental to the success of the migratory cycle of many shorebirds [23].

Ongoing urbanization of coastal zones may have detrimental effects on the survival, behavior, presence, and abundance of these birds [24–28]. On the global scale, the available data indicate that of the 511 known migratory shorebird populations, 70 % have decreased, whereas only 20 % have increased in number [29]. In Brazil, populations of certain Nearctic migratory shorebirds have been declining [30, 31], which has been attributed primarily to habitat loss and a decline in the availability of feeding resources because of increasing human occupation of coastal zones as well as other activities [14, 15].

In this context, fishing communities can provide important insights into the natural population dynamics of certain species because these populations have detailed knowledge of local ecosystems because of their systematic exploitation of natural resources [32]. Fishing colonies, or associations established to integrate the social, cultural, and economic lives of artisanal fishing communities, can act as important sources of information on the biological characteristics of the areas they exploit [33, 34], and in many cases, they may represent the only source of reliable historical data on local ecosystems [35].

The local ecological knowledge (LEK) of artisanal fishermen may serve to augment scientific data, fill in gaps of available information on a given region [36], and provide a basis for alternative approaches to gathering data. A number of studies have found considerable variation in LEK based on the age or sex of the respondents [37, 38]. Kai et al. [39] concluded that older individuals are able to accumulate a larger body of information over time through memories of their observations and traditions, and Diegues and Saénz-Arroyo et al. [40, 41] also found that older informants had a better understanding of local conditions. Studies of the fishermen along the coast of São Paulo and Rio de Janeiro, Brazil [42] as well as communities in Indonesia [43] also found a positive association between local knowledge and age.

Although a limited number of studies [44–46] have focused on the LEK of artisanal fishermen in relation to migratory shorebirds, most of the research has focused on the coastal areas of North America [47–49]. Studies of the perceptions of artisanal fishermen with regard to the abundance of these birds [50–52] and the effects of human activities on their populations [53] are of considerable interest for the development of effective conservation and management plans for overwintering sites [54] as well as the maintenance of viable populations.

Based on these considerations, the goal of the present study was to examine the perceptions of a population of artisanal fishers in relation to the migratory shorebirds found within their local area and determine the factors that may lead to perceived fluctuations in the size of the shorebird populations. This study also evaluated potential variations in the LEK according to the sex and age of the individual, the type of fishery activities conducted by the individuals, and the areas in which they work. Six main questions were addressed: (i) Do the artisanal fishers have reliable knowledge of migratory shorebirds and the areas in which they can be found? (ii) Have the artisanal fishers perceived any increases or decreases in the populations of migratory shorebirds over time? (iii) Have the artisanal fishers perceived any changes in the landscape over the same period, and if so, (iv) what changes have occurred? (v) Do these changes interfere with the populations of migratory shorebirds? (vi) What practice(s) may interfere with the presence of migratory shorebirds?

## Methods

### Study area

**Fig. 1:**
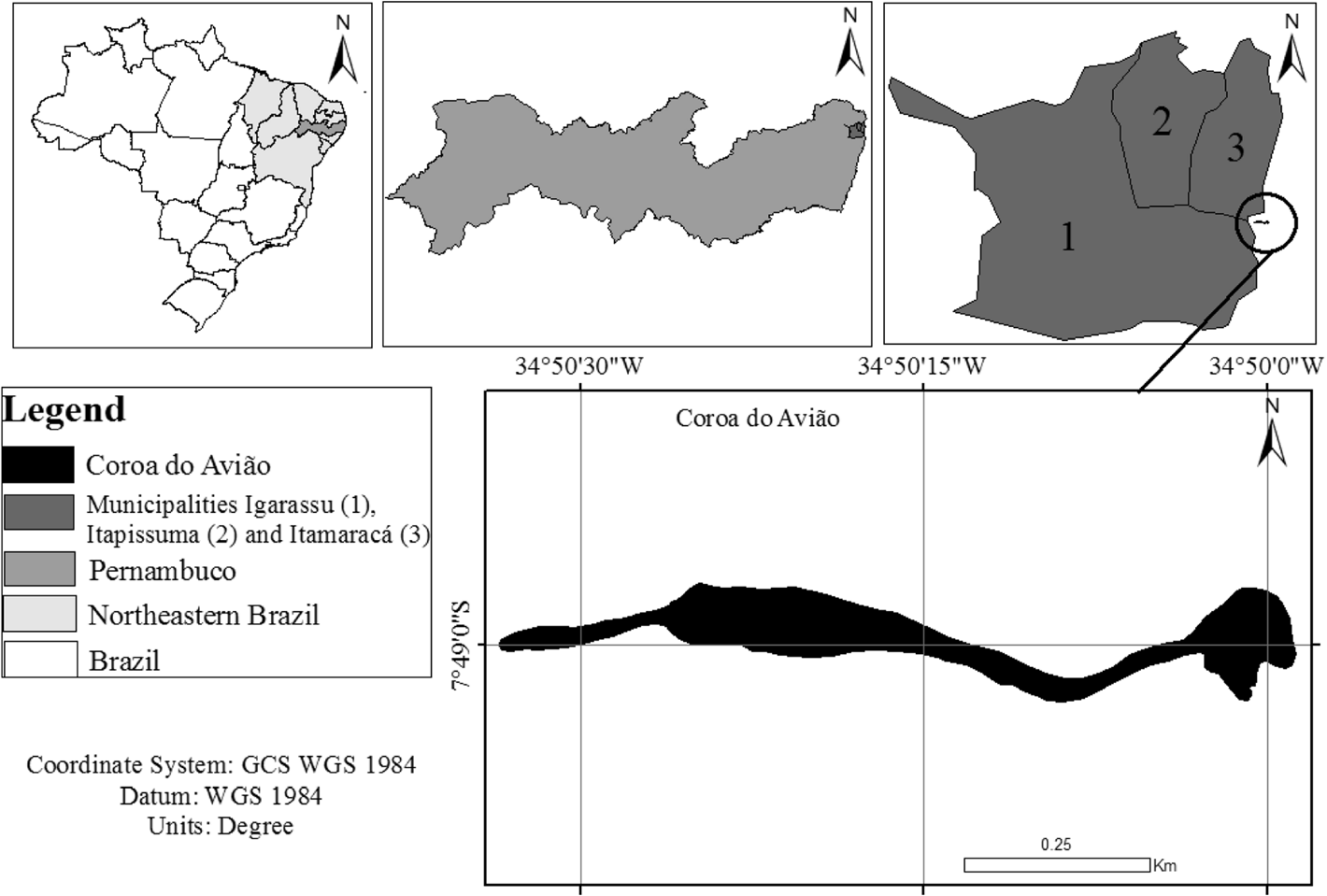
Location of Coroa do Avião Island and the municipalities of Igarassu, Itapissuma and Itamaracá on the southern bar of the Santa Cruz Channel in Igarassu, Pernambuco (NE Brazil)

The present study focused on Coroa do Avião Island, which is located on the southern bar of the Santa Cruz Channel (7°49’00” S, 34°50’15” W), north of Maria Farinha Beach and south of Itamaracá Island on the northern coast of the Brazilian state of Pernambuco [19] (Figure 1). This island is part of the region’s coastal zone and one of the most important overwintering sites for migratory shorebirds in northeast Brazil [18–20, 45, 55].

The island is also frequented by local residents, who harvest mollusks and crustaceans, as well as by tourists, who visit the local bars and restaurants and take boat rides [56]. The livelihoods of approximately 4000 residents of the municipalities of Itapissuma, Igarassu and Itamaracá are dependent on the artisanal exploitation of local fishery resources, which are responsible for more than half the state’s production and form the principal fishery complex of Pernambuco [57].

### Procedures

Data were collected between August 2013 and October 2014 during visits to the artisanal fishing communities registered in the Z-20 fishing colony in Igarassu (7°50’00” S, 34°54’30” W), the Z-10 colony in Itapissuma (07°46’6” S, 34°53’27” W), and the Z-11 colony in Itamaracá (07°45’00” S, 34°49’30” W). These three municipalities are all located on the northern coast of Pernambuco [57–59] (Fig. 1) and were selected because of their proximity to Coroa do Avião Island and the use of their local natural resources by the fishermen.

Most of the members of colony Z-20 are cocklers, or harvesters of the mollusk *Anomalocardia brasiliana* (Gmelin, 1791) (Bivalvia, Veneridae), whereas members of colony Z-10 fish the estuarine habitats of the Santa Cruz channel. Most of the members of colony Z-11 fish the open sea [58, 59].

The only inclusion criterion for the interviewees was membership in one of the study colonies. The fishing colonies were selected because they represent locations where the local fishers are socially and collectively organized; thus, they provide ideal conditions for the development of the present study and subsequent follow-up investigations [60].

The sample size was determined using the statistical approach developed by Arkin and Colton [61], in which populations of fewer than 1000 individuals require the application of 222 questionnaires to ensure that the margin of error is no greater than a 5 %. Here, 240 fishermen were interviewed out of a total of 987 who fit the criterion (Fishermen = 138; Fisherwomen = 102; < 40 years old = 96; > 40 years old = 144; Z20 = 126; Z10 = 73; Z11 = 41). The sampling was non-random and intentional [62] because the informants were defined a priori as members of the Igarassu, Itapissuma, and Itamaracá fishing colonies. The interview questions focused on the participants’ knowledge of the local migratory shorebirds (Table 1) and the population fluctuations of these birds within the study area over time.

**Table 1:** List of migratory and resident birds encountered on Coroa do Avião Island, northeastern Brazil

Scientific name	Popular name	Status
Charadriiformes Huxley, 1867		
Charadrii Huxley, 1867		
CharadriidaeLeach, 1820		
*Pluvialis squatarola* (Linnaeus, 1758)	Black-bellied Plover	Migratory
*Charadrius semipalmatus* Bonaparte, 1825	Semipalmated Plover	Migratory
*Charadrius wilsonia* Ord, 1814	Wilson’s Plover	Resident
*Charadrius collaris* Vieillot, 1818	Collared Plover	Resident
Haematopodidae Bonaparte, 1838		
*Haematopus palliatus Temminck, 1820*	American Oystercatcher	Resident
Scolopaci Steijneger, 1885		
Scolopacidae Rafinesque, 1815		
*Limnodromus griseus* (Gmelin, 1789)	Short-billed Dowitcher	Migratory
*Numenius hudsonicus*Latham, 1790	Hudsonian Whimbrel	Migratory
*Actitis macularius* (Linnaeus, 1766)	Spotted Sandpiper	Migratory
*Tringa solitaria* Wilson, 1813	Solitary Sandpiper	Migratory
*Tringa melanoleuca* (Gmelin, 1789)	Greater Yellowlegs	Migratory
*Tringa semipalmata* (Gmelin, 1789)	Willet	Migratory
*Tringa flavipes* (Gmelin, 1789)	Lesser Yellowlegs	Migratory
*Arenaria interpres* (Linnaeus, 1758)	Ruddy Turnstone	Migratory
*Calidris canutus* (Linnaeus, 1758)	Red Knot	Migratory
*Calidris alba* (Pallas, 1764)	Sanderling	Migratory
*Calidris pusilla* (Linnaeus, 1766)	Semipalmated Sandpiper	Migratory
*Calidris minutilla* (Vieillot, 1819)	Least Sandpiper	Migratory
*Calidris fuscicollis* (Vieillot, 1819)	White-rumped Sandpiper	Migratory
Laridae Rafinesque, 1815		
*Chroicocephalus cirrocephalus* (Vieillot, 1818)	Grey-hooded Gull	Resident
SternidaeVigors, 1825		
*Sternula antillarum*Lesson, 1847	Least Tern	Migratory
*Gelochelidon nilotica* (Gmelin, 1789)	Gull-billed Tern	Resident
*Sterna hirundo*Linnaeus, 1758	Common Tern	Migratory

### Data analysis

To analyze potential differences in the fishers from the different colonies in relation to fishing activities, age (over and under 40 years of age) and sex (male/female), the data were analyzed using Chi-square test, G-test, and Kruskal-Wallis ANOVA, and contingency tables were constructed in Microsoft Excel. All of the analyses were run in BioEstat 5.0 [63], and a significance level of *p* ≤ 0.05 was used for all of the analyses. Only the confirmatory responses (negative/positive) were included in the analysis, and responses in which the interviewee failed to provide specific information (“don’t know”/“no opinion”) were discarded.

## Results

A majority of the male informants (62.9 %, *n* = 78) affirmed that they could identify migratory birds, whereas only 37.1 % (*n* = 46) of the female informants responded positively (Table 2). Based on these values, the male respondents were significantly more knowledgeable than the females with regard to this specific topic (*χ*^2^ = 3.919; d.f. = 1; *p* = 0.0477). The participants that were less than 40 years old (68.9 %; *n* = 73) were also significantly more knowledgeable (*χ*^2^ = 18.651; d.f. = 1; *p* = 0.0001) than the older participants (Table 2). No significant difference was found among the three fishing colonies, however.

**Table 2:** Perception of the fishers in the region of Coroa do Avião Island with regard to the local population of migratory shorebirds

Question	Answer	Gender		Age (%)		Colony			Test
		M%	F%	40 %	40 + %	IT%	IP%	IG%	
Do you know what shorebirds are?	no	40.9	54.0	31.1	59.5	38.5	43.7	50.8	Gender: (*χ* ^2^ = 3.919; n = 232; d.f. = 1; p = 0.0477) Age: (*χ* ^2^ = 18.651; *n* = 232; d.f. = 1; *p* = 0.0001) Colony: (*χ* ^2^ = 2.157; *n* = 232; d.f. = 2; *p* = 0.34)
	yes	59.1	46.0	68.9	40.5	61.5	56.3	49.2	
Do you know the areas where shorebirds can be found?	no	21.6	46.7	37.3	29.1	54.1	20.0	32.2	Gender: (*χ* ^2^ = 15.326; *n* = 217; d.f. = 1; *p* = 0.0001) Age: (*χ* ^2^ = 1.594; *n* = 217; d.f. = 1; *p* = 0.2067) Colony: (*χ* ^2^ = 12.514; *n* = 217; d.f. = 2; *p* = 0.0019)
	yes	78.4	53.3	62.7	70.9	45.9	80.0	67.8	
Are these birds faithful to these sites?	SS	50.5	61.5	45.5	58.7	54.5	45.5	59.8	Gender: (*χ* ^2^ = 1.931; *n* = 159; d.f. = 2; *p* = 0,3807) Age: (*χ* ^2^ = 2.971; *n* = 159; d.f. = 2; *p* = 0.2264) Colony: (G = 9.3748; *n* = 159; d.f. = 4; *p* = 0.0524)
	DS	12.1	11.5	16.4	9.6	27.3	9.1	9.8	
	NS	37.4	26.9	38.2	31.7	18.2	45.5	30.5	
What causes this behavior?	LC	21.0	8.9	4.8	22.3	12.5	25.9	11.6	Gender: (G = 4.1926; *n* = 145; d.f. = 2; *p* = 0.1229) Age: (G = 14.583; *n* = 145; d.f. = 2; *p* = 0.0007) Colony: (G = 7.1067; *n* = 147; d.f. = 4; *p* = 0.1304)
	HT	76.0	84.4	83.3	76.7	83.3	72.2	79.7	
	ED	3.0	6.7	11.9	1.0	4.2	1.9	8.7	
Have their flocks decreased in size?	No	46.8	43.2	40.0	42.9	43.3	39.1	41.2	Gender: (χ = 1.724; *n* = 194; d.f. = 1; *p* = 0.1892) Age: (*χ* ^2^ = 0.199; *n* = 194; d.f. = 1; *p* = 0.6558) Colony: (*χ* ^2^ = 0.322; *n* = 194; d.f. = 2; *p* = 0.8512)
	Yes	53.2	56.8	60.0	57.1	56.7	60.9	58.8	
Do you perceive any changes in the island?	No	21.4	37.7	29	27.5	21.2	22.6	33	Gender: (*χ* ^2^ = 5.959;*n* = 189;d.f. = 1;*p* = 0,0146) Age: (*χ* ^2^ = 0.048; *n* = 189; d.f. = 1; *p* = 0.8267) Colony: (*χ* ^2^ = 3.357; *n* = 189; d.f. = 2; *p* = 0.1867)
	Yes	78.6	62.3	71	72.5	78.8	77.4	66	
What changes are these?	HB	58.7	57.4	53.3	61.0	50.0	56.1	61.9	Gender: (*χ* ^2^ = 0.218; *n* = 252; d.f. = 2; *p* = 0.8969) Age: (*χ* ^2^ = 0.839; *n* = 252; d.f. = 2; *p* = 0.6574) Colony: (G = 5.7475; *n* = 122; d.f. = 4; *p* = 0.2188)
	CE	25.3	23.4	28.9	22.1	44.4	24.4	19.0	
	DE	16.0	19.1	17.8	16.9	5.6	19.5	19.0	
What changes have provoked a reduction in shorebird populations?	1	19.2	11.6	13.0	18.5	12.5	20.9	15.0	Gender: (*χ* ^2^ = 4.807; *n* = 199; d.f. = 5; *p* = 0.4399) Age: (*χ* ^2^ = 5.474; *n* = 199; d.f. = 5; *p* = 0.3608) Colony: (H = 10.7232; *n* = 199; d.f. = 2; *p* = 0.0047)
	2	13.1	10.1	15.9	10.0	3.1	11.9	15.0	
	3	18.5	15.9	13.0	20.0	12.5	20.9	17.0	
	4	23.8	36.2	34.8	24.6	43.8	22.4	27.0	
	5	10.8	13.0	11.6	11.5	3.1	10.4	15.0	
	6	14.6	13.0	11.6	15.4	25.0	13.4	11.0	
Which practices interfere with the presence of the birds?	7	13.8	13.2	16.8	11.8	14.6	12.8	13.8	Gender: (*χ* ^2^ = 3.958; *n* = 279; d.f. = 4; *p* = 0.4117) Age: (*χ* ^2^ = 2.571; *n* = 279; d.f. = 4; *p* = 0.632) Colony: (H = 9.5461; *n* = 279; d.f. = 2; *p* = 0.0085)
	8	24.5	25.3	25.7	24.2	16.7	29.1	24.8	
	9	23.4	17.6	22.8	20.8	18.8	24.4	20.7	
	10	14.4	23.1	14.9	18.5	27.1	10.5	17.9	
	11	23.9	20.9	19.8	24.7	22.9	23.3	22.8	

M%: Percentage of male informants; F%: Percentage of female informants; −40 %: Percentage of informants less than 40 years of age; 40 + %: Percentage of informants older than 40 years of age; IT%: Percentage of the fishers from Itamaracá; IP%: Percentage of fishers from Itapissuma; IG%: Percentage of fishers from Igarassu

*Abbreviations*: *SS* same sites, *DS* same sites and different sites, *NS* new sites, *LC* life cycle of the birds, *HT* habitat/environment, *ED* environmental degradation, *HB* housing boom, *CE* conservation of the environment, *DE* degradation of the environment

1: Hotel Amoaras/Gavôa; 2: Airfield; 3: Shrimp farms; 4: Housing boom; 5: Construction of factories; 6: Other; 7: Ultralight planes, helicopters and airplanes flying overhead; 8: Boat traffic around the island; 9: Movements of tourists on the island; 10: Accumulation of garbage on the island; 11: Sound pollution from bars and boats

The male informants were also significantly more knowledgeable (*χ*^2^ = 15.326; d.f. = 1; *p* = 0.0001) than the female informants with regard to the areas in which the birds can be observed, although in both cases, the percentage of positive responses increased, with values of 78.4 % (*n* = 98) among male respondents and 53.3 % (*n* = 49) among female respondents. Similarly, a higher percentage of younger informants (70.9 %, *n* = 95) confirmed that they knew the location of the birds’ resting and foraging sites compared with those older than 40 years of age (62.7 %, *n* = 52), although this difference was not significant. However, a significant difference (*χ*^2^ = 12.514; d.f. = 2; *p* = 0.0019) was observed among the fishing colonies with regard to the birds’ resting and foraging sites, with informants from Z20 (80.0 %; *n* = 78) and Z10 (67.8 %; *n* = 52) claiming more knowledgeable on this question compared with those from Z11.

With regard to the fidelity of the birds to their overwintering sites, 50.5 % (*n* = 54) of the male informants confirmed seeing the birds at the same sites, whereas 61.5 % (*n* = 32) of the female informants responded positively to the same question, although this difference was not statistically significant. Similarly, although a higher percentage of older informants (58.7 %; *n* = 61) confirmed seeing the birds in the same area compared with the younger informants (45.5 %, *n* = 25), the difference was not statistically significant. The informants from Z20 and Z10 provided a greater number of references to the site fidelity of the birds compared with those from Z11 (Table 2), although the difference among the colonies did not reach significance (G = 9.3748; *n* = 159; d.f. = 4; *p* = 0.0524).

With regard to the participants’ beliefs regarding the causes underlying the use of new areas by the birds for foraging and resting, 76.0 % (*n* = 76) of the male informants and 84.4 % (*n* = 38) of females referred to habitat-related factors as the determinants of birds occupying a given area, although the difference between the sexes was not significant. Between age groups, a significant difference was observed in relation to the importance of habitat as the principal determinant of the permanence of the birds in a given area (G = 14.583; *n* = 145; d.f. = 2; *p* = 0.0007). Environmental degradation was the second-most important factor among younger informants (11.9 %; *n* = 5), whereas the life cycle of the birds (foraging, resting) was the second-most important factor among older informants (22.3 %; *n* = 23); however, significant variations were not observed among the colonies (Table 2).

Significant differences were not observed between age groups or between genders in relation to the participants’ beliefs regarding the reduced populations of migratory shorebirds (Table 2), and in all groups, a majority of informants indicated that a reduction in the population of birds on the island had occurred over time (male: 62.6 %; female: 53.2 %; < 40 years old: 56.8 %; ≥ 40 years old: 60.0 %).

Most (78.6 %; *n* = 88) of the male informants believe that Coroa do Avião Island has changed, although a smaller percentage of the female informants (62.3 %; *n* = 48) held this view, with the difference reaching significance (*χ*^2^ = 5.959; *n* = 189; d.f. = 1; *p* = 0.0146); however, significant differences were not observed between age groups (Table 2). The male and female participants from Z20 were the least likely to state that changes had occurred on the island, although the differences among the three colonies with regard to this question were not significant (Table 2).

The vast majority of the informants were unanimous in blaming the recent landscape modifications on the local housing boom (Table 2), which has also resulted in an increase in the number of people occupying the area, and these results were irrespective of the sex (*χ*^2^ = 0.018; *n* = 122; d.f. = 1; *p* = 0.8942), age (*χ*^2^ = 0.693; *n* = 122; d.f. = 1; *p* = 0.4051) or colony membership (*χ*^2^ = 0.927; *n* = 122; d.f. = 2; *p* = 0.6289) of the informant.

When asked to identify the principal cause of the reduced number of migratory shorebird numbers on Coroa do Avião Island, 23.8 % (*n* = 31) of the male informants and 36.2 % (*n* = 25) of the female informants referred to the local housing boom, whereas 19.2 % (*n* = 25) of the male informants referred to the construction of hotels in the region as the principal factor, and 15.9 % (*n* = 11) of the female informants cited the development of shrimp farms, although significant differences were not observed between the sexes overall. Similarly, significant difference were not observed with regard to the informants’ responses based on age, with 34.8 % (*n* = 24) of the younger informants and 24.6 % (*n* = 32) of the older informants citing the local housing boom as the principal cause of the reduction in shorebird populations. However, although 43.8 % (*n* = 14) of the fishermen from Z11 and 27.0 % (*n* = 27) of the fishers from Z20 cited the housing boom as the principal factor affecting bird populations (Table 2), those from Z10 primarily blamed the construction of hotels and shrimp farms, which resulted in a significant difference among colonies (H = 10.7232; *n* = 199; d.f. = 2; *p* = 0.0047).

**Fig. 2:**
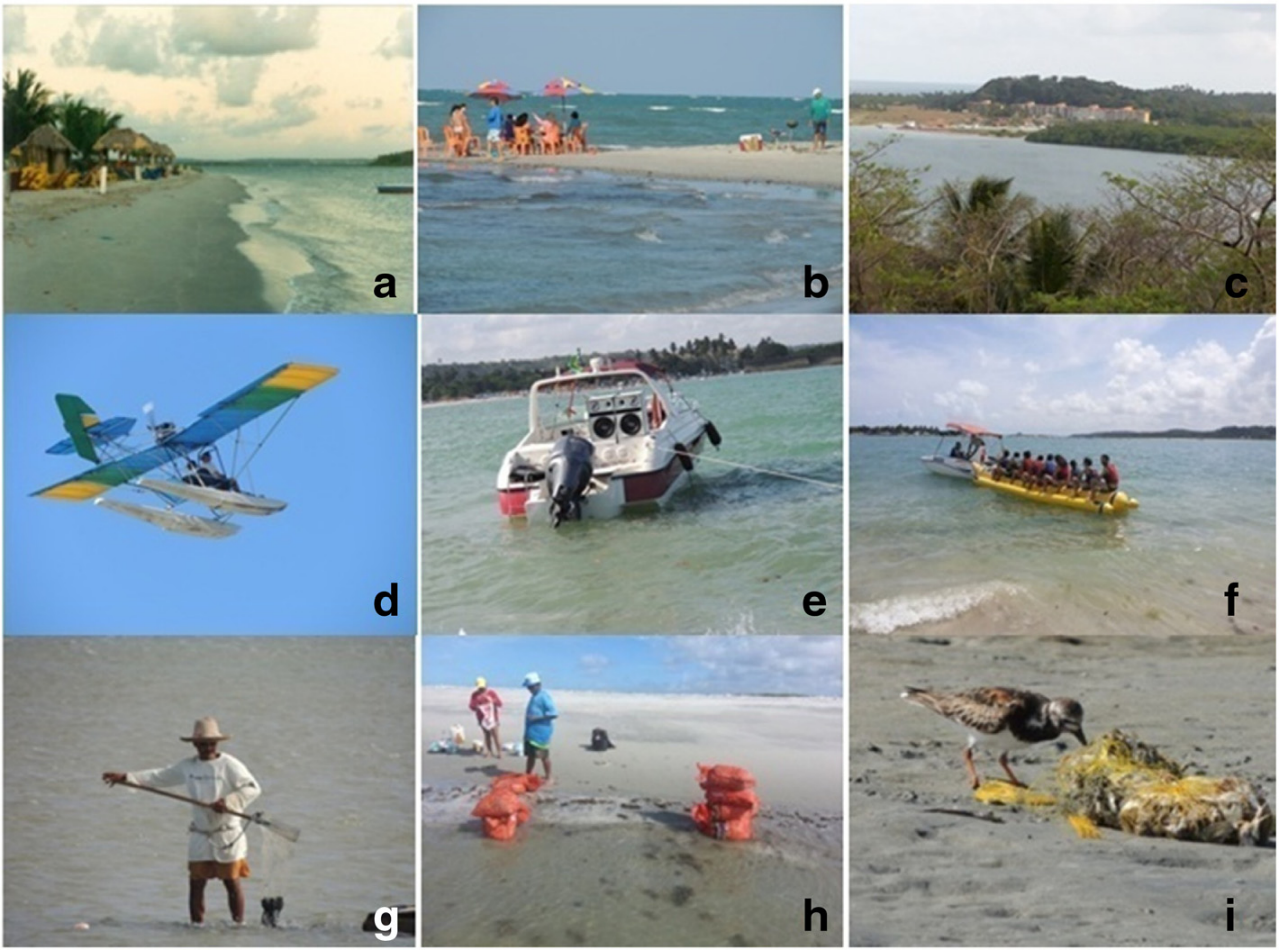
Examples of the impacts that have contributed to the reduced numbers of shorebirds on Coroa do Avião Island according to the perception of local fishers from Z20, Z11 and Z10, Pernambuco (NE Brazil). **a, b, c** Increasing number of bars and tourism infrastructure; (**d**, **e**, **f**) sound pollution from boats and ultralight aircraft; (**g**, **h**) increased harvesting of shellfish; (I) garbage. (**a**, **b**) Bars and restaurants with the presence of tourists; (**c**) hotel; (**d**, **e**, **f**) ultralight aircraft and tourist boats; (**g**, **h**) cocklers harvesting mollusks; (**i**) shorebird on the beach in the vicinity of garbage. Photographs: Telino-Júnior WR. 2013–2014 (**a**, **b**, **d**, **e**, **g**, **h**, **i**); Andrade LP. 2014 (**c**, **f**)

The informants referred to a wide variety of practices that may have an effect on the presence of shorebirds on Coroa do Avião Island (Fig. 2). Boat traffic around the island was the principal practice cited by both the male (24.5 %; *n* = 46) and female (25.35 %; *n* = 23) informants. However, the second-most common practice named by males (23.9 %; *n* = 44) was noise pollution from bars and boats, whereas the female informants pointed to the accumulation of garbage on the island (20.9 %; *n* = 21). However, the difference between the sexes was not significant (Table 2).

Significant differences were observed among the colonies with regard to the factors that have an effect on the presence of shorebirds (H = 9.5461; *n* = 279; d.f. = 2; *p* = 0.0085). The informants from Z10 (29.1 %; *n* = 25) and Z20 (24.8 %; *n* = 36) referred primarily to boat traffic and tourist activities (24.4 %; *n* = 21 and 20.7 % *n* = 30, respectively), whereas the informants from Z11 referred primarily to the accumulation of garbage on the island (27.1 %; *n* = 13) and noise pollution from boats and bars (22.9 %; *n* = 11).

## Discussion

The fishers interviewed for this study were relatively knowledgeable about the bird species found in their local area. Because these individuals depend on natural resources for their survival, they were expected to have a certain amount of empirical knowledge of and experience with the biological and ecological characteristics of the local fauna and flora. A similar situation was recorded by Silvano et al. [64] in a study of fish migration and reproduction on the northeastern and southeastern coasts of Brazil and by Zappes et al. [65] in their investigation of the interaction between the common bottlenose dolphin, *Tursiops truncates*, and fisheries in Brazil and Uruguay.

The observed differences between the male and female respondents appears to be related to the greater mobility of men during the fishery activities practiced within the study area. Kai et al. [39] also found that men had greater local ecological knowledge compared with women, which was likely because the male participants were more familiar with the studied animals. The higher level of knowledge regarding shorebirds observed in the present study by the younger informants is inconsistent with the results of previous studies [64, 66, 67], which found a direct relationship between age or experience and local knowledge. Shen et al. [68] concluded that improvements to formal education and schooling levels contributed to increases in scientific ecological knowledge and promoted a better understanding of local environments. In this context, the greater knowledge of birds by the younger informants reported here may have been related to the environmental education programs organized by the Federal Rural University of Pernambuco (and coordinated by two authors of this paper) on Coroa do Avião Island (Ecotourism Museum) in partnership with the municipal authorities of Z20, Z10 and Z11, including the region’s municipal schools. The target population of these programs included children and adolescents who are now young adults of less than 40 years of age; however, this hypothesis remains to be tested.

The greater familiarity of male informants with the resting and foraging areas of the shorebirds was likely related to local traditions in which sons accompany their fathers during fishing trips from a young age and thus accumulate local ecological knowledge much sooner than their female peers. Similar situations were observed by Kai et al. [39] and Alves et al. [69]. In turn, the significant differences observed among the colonies appeared to be related to differences in their fishery practices influencing the perception of natural resources by the respective fishers, which has also been reported in previous studies [36, 67]. In particular, the daily routine of the fishermen from Z10 and Z20 provided more frequent contact with the areas in which the migrating shorebirds are found.

With regard to the migratory shorebirds’ fidelity to specific overwintering sites, the frequency of responses referring to the colonization of new sites by the birds for foraging and resting reinforces the need for further research because these birds are known to be faithful to their foraging sites [70, 71]. The apparent use of new areas may reflect transformations that have occurred to their original feeding grounds; thus, it is important to identify and quantify these alterations. A characteristic behavior of migratory shorebirds is their ability to respond rapidly to anthropogenic disturbances [72], which means that the behavior perceived by the male and female participants may indicate adverse local conditions as observed in previous studies [24–27].

The fishers referred to the life cycle of the birds and their requirements for feeding and resting areas as the principal factors driving the birds’ search for new areas, and these results are similar to those of previous studies [25, 73–76]. Atkinson et al. [77] concluded that a reduction in the populations of invertebrates that compose the principal diets of migratory shorebirds would result in a decline in the population of shorebirds, and this conclusion is consistent with the observations of the participants in the present study, who stated that the birds explore new foraging areas in search of feeding resources, which the fishermen associated with the increased harvesting of bivalve mollusks (*A. brasiliana*). Such harvests have resulted in the progressive decline in the quantity and quality (size) of these mollusks with each passing year. Because the birds also feed on these mollusks [22], there is a clear link between decreasing mollusk populations caused by overharvesting and the birds’ search for new and better feeding grounds. In this context, the quality of overwintering sites is fundamental to the successful conclusion of the shorebirds’ migratory cycle because of their need to accumulate energetic reserves to fuel the long journey back to the Northern Hemisphere in the boreal spring for the subsequent breeding season [78, 79].

Gill et al. [80], Rodgers Jr. and Schwikert [81], and Burger et al. [26] also observed that shorebirds explored new foraging areas because of the effects of human disturbance, which in most cases was related to the loss of environmental quality, particularly the availability of feeding resources [82]. Such changes will force migratory birds, even if temporarily, to find new less-disturbed areas with higher-quality food resources.

Based on the perspective of the local inhabitants as well as the results of recent studies in the area, the decline in food resources may be related to progressive increases in industrial and domestic effluents, including heavy metals and runoff of agricultural chemicals used on the plantations located in the surrounding area [83], which are transported by waterways that discharge into the estuaries of the Santa Cruz Channel. With regard to the specific area covered in this study, the informants referred to the recent construction of several industrial plants in the region, the development of shrimp farms and the increasing use of pesticides on local sugarcane plantations as the principal factors contributing to the environmental degradation of the region.

The significant influence of age on the informants’ perceptions of environmental degradation, including the pollution and destruction of mangroves and the life cycle of shorebirds, may be related to their lack of scientific knowledge with regard to the environmental changes occurring in the area and a failure to transmit this information adequately between generations, thus resulting in differences in the perception of these processes [84–86]. Based on this perspective, Pauly [87] introduced the “shifting baseline syndrome” (SBS), which proposes that people have difficulty perceiving environmental modifications that have occurred over time as well as previous ecological conditions. According to this theory, the frame of reference of the fishers interviewed in this study for the size of a bird population would correspond to their perception of the population size at the start of their fishing activities and not to the actual abundance of birds at that moment, which may result in an underestimation of losses [88]. In such cases, as one generation is replaced by the next, the references of the fishermen are altered [41], thus resulting in a shift in the baseline perceptions of that population [87].

The perception of the fishers with regard to fluctuations in the populations of migratory shorebirds is based on observations and experiences acquired over time through their contact with the local environment, which was also noted by Yli-Pelkonen and Kohl [89]. This finding is consistent with the survey data from the study area, which show that the populations of certain bird species have been declining since the 1990s [19]. These fluctuations in bird populations have also been observed on Coroa do Avião Island [45] and the coast of southeastern Brazil [44]. This variation may be related to the greater or lesser capacity of different migratory shorebird species to adapt to new conditions or changes in the quality of their overwintering sites [47, 48].

The greater knowledge of male informants than female informants with regard to changes in the local landscape over time was similar to the pattern observed by Begossi [42] and Hanazaki [38]. However, differences were not observed with regard to the age of the informants, and this result may be related to the awareness of these alterations by individuals of all ages are aware, which is likely because these changes have a direct impact on their productivity and, ultimately, their income.

Although differences were not observed among the three colonies with regard to the factors underlying change to the landscape, there was a broad consensus that the changes in the local landscape and their effects on the bird population were the result of human actions. The perceptions of local fishermen were confirmed by Dryer in Alberta, Canada [90]; LeDee et al. [91] and Drake et al. [92] in the Gulf of Mexico; and Norris [93] in Europe, the United States and Caribbean, with their results showing that the loss of habitat quality is reflected in a reduction in the bird populations that use the area. Sutherland [94] concluded that the greatest challenge with regard to seabirds is gaining a better understanding of the amplitude of the cumulative impacts of relatively minor changes and disturbances to the environment over time.

With regard to the changes in the local landscape, the almost significant difference found between the fishing colonies of Z11 and Z20 may be related to the fact that the fishermen in the latter group spend far more time in the study area because their fishing practice expose them more to estuarine environments rather than sea environments. Coastal beaches and mangroves experienced ever-increasing pressure from human populations throughout the 20^th^ century and into the 21^st^ century [95] because of urbanization and industrial and agricultural development, and these processes were clearly perceived by the local fishermen.

Over the past several decades, government incentives to develop the tourism industry have stimulated a housing boom in the region. The disturbance of overwintering sites by the increased number of people and domestic animals is another possible cause of the decline in the populations of migratory birds [44, 45], and this situation is reinforced by the strong pressure from the tourism industry in the present study area [49]. Moreover, the participants provided consistent responses with regard to their belief that the housing boom along with other factors had a combined effect on the reduction of bird populations.

However, considerable variation was observed in the participants’ responses with regard to the practices that may be interfering with the presence of shorebirds on Coroa do Avião Island. Boat and aircraft traffic and the burgeoning tourist industry were all cited by the informants in the present study and have also been recorded in previous studies. Oliveira et al. [82] and Cardoso and Nascimento [45] considered that all of the practices identified by the informants in their studies had an impact on the conservation of the habitats frequented by migratory birds, and Galbrath [95] and Evans [96] also demonstrated that anthropogenic pressures on overwintering sites reduces the quality of the sites and affects the bird populations in those areas.

In a study on the sensitivity of migratory birds to disturbances caused by boats in the Bay of Fundy, which is on the east coast of Canada, Ronconi and St. Clair [97] concluded that the degree of disturbance is primarily related to the speed of the vessel rather than its size as well as the height of the tide. Cardoso and Nascimento [45] observed a similar pattern of disturbance of shorebird populations resulting from tourist activities. The construction of an airfield in Nova Cruz, Igarassu, which is close to Coroa do Avião Island, has resulted in airplanes flying over the study area and disturbing foraging birds, which was predicted by Sutherland [94]. Cardoso and Nascimento [45] also confirmed the occurrence of sound pollution from bars located on the west side of the islands as well as from boats, with the disturbance principally occurring during weekends. In South Carolina (USA), Peters and Otis [98] found that certain species of migratory birds are highly sensitive to human disturbance and prefer to remain at distances of more than 1000 m from areas with large numbers of boats or people.

Sandy tropical beaches are key habitats for the conservation of migratory shorebirds [95]; however, they are also attractive sites for tourism development and recreational activities [45], which have a major impact on local populations of shorebirds regardless of the extent and timing of these activities [45].

In many cultures, migratory birds are perceived to be sentinels of change [99], and access to the local ecological knowledge of fishers can provide indicators of ecological change that is currently under way as well as the potential motivating factors. This information may be incorporated into action plans and management strategies for the conservation of bird populations and local environments [100, 101]. Research conducted in the Solomon Islands [102], Belize [103], and Hawaii as well as in other areas of the Pacific [104] demonstrate the effectiveness of using local knowledge in the implementation of successful management actions because such use reflects the vast knowledge that has been acquired and accumulated by local people over many years through access and use of their local natural resources [see 105].

## Conclusions

We found that most of the fishers attribute the decreased populations of migratory shorebirds to landscape modifications related to the expansion of industry and local tourism. The local ecological knowledge constitutes an important source of largely untapped information, and such knowledge can be used to test new hypotheses designed to provide more effective conservation actions for bird diversity.

## Ethics approval

An outline of the intended research project and the informed consent forms (ICFs) of the interviewees were submitted online to the Ethics Committees prior to the initiation of the study: Brazilian Committee on Human Ethics (Platform Brazil) and Ethics Committee of Pernambuco State University (UPE), registered under number 30734313.0.0000.5207. The research began after receiving the approval of these committees.

## Consent to participate

The fishers were selected, informed of the research objectives and asked to provide consent prior to their participation. Subsequent to their verbal consent, the fishermen who agreed to participate were invited to read, complete, and sign an informed consent form (ICF). The research was initiated only upon receipt of a signed ICF by the interviewees.

## Consent for publication

The authors declare that they consent to publish this article.
